# Immune Response on Optimal Timing and Fractionation Dose for Hypofractionated Radiotherapy in Non–Small-Cell Lung Cancer

**DOI:** 10.3389/fmolb.2022.786864

**Published:** 2022-01-24

**Authors:** Xianlan Zhao, Jixi Li, Linpeng Zheng, Qiao Yang, Xu Chen, Xiewan Chen, Yongxin Yu, Feng Li, Jianxiong Cui, Jianguo Sun

**Affiliations:** ^1^ Cancer Institute, Xinqiao Hospital, Army Medical University, Chongqing, China; ^2^ Department of Ultrasound, The 941st Hospital of the PLA Joint Logistic Support Force, Xining, China; ^3^ Department of Basic Medicine, Army Medical University, Chongqing, China

**Keywords:** lung cancer, immune checkpoint inhibitor, hypofractionated radiotherapy, tumor immune microenvironment, dynamic changes

## Abstract

**Background:** The intervention timing of immune checkpoint inhibitors (ICIs) and radiotherapy fractionations are critical factors in clinical efficacy. This study aims to explore dynamic changes of the tumor immune microenvironment (TIME) after hypofractionated radiotherapy (HFRT) at different timepoints and fractionation doses in non–small-cell lung cancer (NSCLC).

**Methods:** In the implanted mouse model, the experimental groups received HFRT 3.7 Gy × 4 F, 4.6 Gy × 3 F, 6.2 Gy × 2 F, and 10 Gy × 1 F, respectively, with the same biological equivalent dose (BED) of 20Gy. Tumor volume and survival time were compared with those of the control group. Flow cytometry was performed to detect immune cells and their PD-1/PD-L1 expressions using tail-tip blood at different timepoints and tumor tissues at 48 h after radiotherapy. In NSCLC patients, immune cells, PD-1/PD-L1, and cytokines were detected in peripheral blood for 4 consecutive days after different fractionation radiotherapy with the same BED of 40Gy.

**Results:** Tumor volumes were significantly reduced in all experimental groups compared with the control group, and the survival time in 6.2 Gy × 2 F (*p* < 0.05) was significantly prolonged. In tail-tip blood of mice, CD8^+^ T counts increased from 48 h to 3 weeks in 4.6 Gy × 3 F and 6.2 Gy × 2 F, and CD8^+^ PD-1 shortly increased from 48 h to 2 weeks in 6.2 Gy × 2 F and 10 Gy × 1 F (*p* < 0.05). Dentritic cells (DCs) were recruited from 2 to 3 weeks (*p* < 0.01). As for NSCLC patients, CD8^+^ T counts and PD-1 expression increased from 24 h in 6.2 Gy × 4 F, and CD8^+^ T counts increased at 96 h in 10 Gy × 2 F (*p* < 0.05) in peripheral blood. DC cells were tentatively recruited at 48 h and enhanced PD-L1 expression from 24 h in both 6.2 Gy × 4 F and 10 Gy × 2 F (*p* < 0.05). Besides, serum IL-10 increased from 24 h in 6.2 Gy × 4 F (*p* < 0.05). Conversely, serum IL-4 decreased at 24 and 96 h in 10 Gy × 2 F (*p* < 0.05).

**Conclusion:** HFRT induces the increase in CD8^+^ T cells and positive immune cytokine response in specific periods and fractionation doses. It was the optimal time window from 48 h to 2 weeks for the immune response, especially in 6.2 Gy fractionation. The best immune response was 96 h later in 10 Gy fractionation, delivering twice instead of a single dose. During this time window, the intervention of immunotherapy may achieve a better effect.

## Introduction

Studies have shown that radiotherapy, especially stereotactic body radiation therapy (SBRT) or hypofractionated radiotherapy (HFRT), can cause DNA damage, which leads to tumor cell death, induces release of pro-inflammatory factors, and enhances tumor immune stimulation cells and cytokines to remodel the tumor immune microenvironment (TIME) (Formenti and Demaria, 2013; Demaria et al., 2015). More importantly, radiotherapy can also promote immune cell infiltration and transform “cold” tumors into “hot” tumors, a status suitable to immune checkpoint inhibitors (ICIs) (Ostrand-Rosenberg and Sinha, 2009). Therefore, the combination of radiotherapy and ICI therapy has gained more and more attention and is considered a promising treatment for cancer (Formenti et al., 2018; Chicas-Sett et al., 2019). Dewan et al. (Dewan et al., 2009) found that the combination of HFRT and ICI therapy could induce an abscopal effect in a mouse model of breast cancer. Besides, Verbrugge et al. (Verbrugge et al., 2012) verified that ICI therapy enhanced the curative capacity of radiotherapy in established breast malignancy.

ICI treatment was given at different timepoints after radiotherapy in many studies; therefore the optimal time window remains elusive (Dovedi et al., 2015; Schapira et al., 2018). The PACIFIC study showed that interventional immunotherapy within 14 days after radiotherapy had the longer progression-free survival (PFS) and overall survival (OS) in patients with locally advanced NSCLC (Antonia et al., 2018). The KEYNOTE-001 study found that radiotherapy followed by immunotherapy had better PFS (4.4 months vs*.* 2.1 months) and OS (10.7 months vs. 5.3 months) in patients with advanced NSCLC (Shaverdian et al., 2017). The Pembro-RT study verified that pembrolizumab within 1 week after SBRT doubled the objective response rate (ORR), and prolonged PFS (6.6 months vs*.* 1.9 months) and OS (15.9 months vs*.* 7.6 months) in patients with advanced NSCLC (Theelen et al., 2019). Bauml’s study revealed that pembrolizumab in 4–12 weeks after local ablations had a PFS of 19.1 months in patients with metastatic NSCLC, tripling the previous PFS of 6.6 months (Aggarwal et al., 2019). However, Wegner (Wegner et al., 2019) showed that immunotherapy at least 3 weeks after radiotherapy would exhibit longer OS in a retrospective study. Therefore, to explore the right timing for ICI therapy intervention after radiotherapy has great significance in clinical treatment.

What is more, different fractionations also have different effects on TIME. Lugade (Lugade et al., 2008) found that a single 15 Gy was more effective than 3 Gy × 5 F in activating DC cells in lymph nodes in the B16 melanoma model. However, Schaue (Schaue et al., 2012) found that the 7.5 Gy × 2 F was better than a single dose of 15 Gy in inducing T cell initiation in another melanoma model. An appropriate fractionation could enhance an immunoreactive effect, but an extra high dose would cause damage to lymphocyte subsets and produce an immunosuppressive effect and immune dysfunction (Zitvogel and Kroemer, 2015). A single high-dose radiotherapy could cause damage and collapse of the tumor vasculature, which was not conducive to the infiltration of T cells into the tumor (Timke et al., 2008). It would cause radioresistance of tumor cells due to hypoxia caused by destruction of the vascular system (Barker et al., 2015). Radiation produces two-way immune effects like the “seesaw,” including positive and negative responses. The appropriate fractionation could push the immune effects into the positive response. Previous studies have shown that HFRT or SBRT was more capable of mobilizing local and systemic immune responses than conventional fractionation (Schaue and Mcbride, 2015). Since there are many choices in clinical practice, it is a conundrum as to which fractionation is appropriate and optimal.

As the intervention timepoints of ICI therapy after radiotherapy and the fractionations are various and controversial in previous studies, this study aimed at exploring the dynamic changes of TIME at different timepoints and fractionation doses of HFRT in NSCLC and providing an experimental basis for the optimal intervention timing and fractionation dose for the combination of radiotherapy and ICI therapy.

## Materials and Methods

### Radiation of Lung Cancer Implanted Mouse Model

#### Mice and Cell Line

A total of 60 C57BL/6 male mice (6–8 weeks old) were purchased from the animal center of our hospital (No. SYXK 2012-0011). All protocols were approved by the Laboratory Animal Welfare and Ethics Committee of Army Medical University (Chongqing, China). Lewis lung carcinoma (LLC) cells were maintained in DMEM culture medium (Gibco, United States) supplemented with 10% fetal bovine serum (HyClone, United States), 100 U/ml penicillin, and 100 μg/ml streptomycin.

#### Lewis Cell Inoculation Into Mouse

1×10^6^ Lewis cells were inoculated subcutaneously to the right leg of the mice. Tumor size was measured using a vernier caliper every 3 days. Tumor volume was calculated as follows: tumor volume (mm^3^) = (long axis) × (short axis) ^2^/2.

#### Irradiation Plans

25 mice were selected with a tumor volume of about 100 mm^3^ and randomly divided into the control group and 4 experimental groups with 5 mice in each group. Experimental groups were anesthetized and given radiotherapy 3.7 Gy × 4 F, 4.6 Gy × 3 F, 6.2 Gy × 2 F, and 10 Gy × 1 F, respectively, using 6MV X-ray with a radiation field of 10 cm × 10 cm. The selection of radiotherapy dose in mice was consistent with a previous study (Mathieu et al., 2021), in which a single dose of 10 Gy induced immune response and even abscopal effects. The four fractionations had the same biological equivalent dose (BED, 20Gy) with the calculation formula BED = nd [1 + d/(ɑ/β)]. Radiotherapy plans were designed using a Varian eclipse treatment planning system (TPS, version 13.5) with the spare of the area of lymph nodes and delivered by the Varian Trilogy Accelerator. The source skin distance (SSD) was 100 cm, the irradiation was at a depth of 0.5 cm, and the dosage rate was 400 MU/min.

Tail-tip blood samples were collected at different timepoints, 1 day before radiotherapy as the baseline and 24 h, 48 h, 96 h, 1 week, 2 weeks, and 3 weeks after finishing radiotherapy. The survival time was observed every 3 days with the following endpoints and given euthanasia: tumor dimension reaching 20 mm, tumor with ulceration, necrosis or infection, and morbility or disability. Another experiment of 20 implanted mice with 4 in each group received the same irradiation, and tumor tissues were collected at 48 h after finishing radiotherapy.

### Clinical Practice

All patients were diagnosed with unresectable stage IV NSCLC by histology or cytology according to the eighth edition of the American Joint Committee on Cancer (AJCC) Union. Other inclusion criteria included 18–75 years old, ECOG performance status 0∼1, and measurable or evaluable lesions. The exclusion criteria included inadequate cardiac, pulmonary, renal, and hepatic functions and blood count/chemistry tests, uncontrolled malignant pleural/pericardial effusions, and previous radiotherapy at the same lesions. We designed the radiotherapy plan based on the NCCN guideline for the lesions. Four plans (3.7 Gy × 8 F, 4.6 Gy × 6 F, 6.2 Gy × 4 F, and 10 Gy × 2 F) with the same BED of 40 Gy were conducted using 6MV X-ray with at least 5 patients in each group. Peripheral blood samples were collected within 1 week before radiotherapy as the baseline and 24, 48, 72, and 96 h after radiotherapy. In clinical practice, BED 40Gy is a better palliative radiotherapy dose than BED 20Gy by NCCN guideline recommendation to relieve symptoms of local lesions. Actually, some patients boost the dose after continuous 4-day blood sample collection to reach the clinical requirement. To better protect the immune system, peripheral draining lymph nodes in mice or patients were not delineated and irradiated as the targets. This study was registered in the Clinical Trials Register (NCT03073902, https://clinicaltrials.gov/). All patients have signed written informed consent forms.

### Tumor Sample Preparation

We collected tumor tissues from implanted mice by cervical dislocation at 48 h after irradiation. Tumor-infiltrating lymphocytes (TILs) were processed by using a gentle Macs dissociator and a murine tumor dissociation kit. Lymphocytes from mice and patients’ anti-freezing blood were obtained with mouse and human peripheral blood lymphocyte isolation fluid (LTS10771, TBD, China). The serum of the NSCLC patient was collected after centrifuging for 10 min at 1,000 rpm.

### Flow Cytometry

The single cell suspension of mouse or human samples was centrifuged at 2500 rpm for 3 min, mixed with CD4 (#100408), CD8 (#100712), Ly-6G/Ly-6C (Gr-1) (# 108412), CD11b (#101208), CD11c (#117306), CD25 (#101908), CD127 (# 135012), CD274 (PD-L1) (#124314), CD279 (PD-1) (#109110) anti-mouse (BD Biosciences, United States) or CD4 (#560650), CD8 (#563256), CD279 (#561787) (R&D system, United States), CD11b (#101228), CD11c (#301624), CD19 (# 302226), CD25 (#302609), CD33 (#303436), CD45 (#304029), HLA-DR (#307616) (Biolegend, Germany), CD274 (#2338640), and CD127 (# 2071281) (Invitrogen, United States) anti-human antibody of immune cells, respectively, after removing the supernatant and then stained at 4°C for 30 min. Dead cells were identified using a LIVE/Dead (LD) immobile dye kit (#1968231, Invitrogen, United States). Data was acquired by multi-parameter flow cytometry (BD Biosciences, United States), and the results were analyzed using FlowJo10.0. Based on the PD1/PD-L1 signaling pathway in tumor immunology (Jiang et al., 2015), we detected the counts of CD4^+^ T cells, CD8^+^ T cells, DC, Treg, and MDSC, the PD-1 expression in circulating immune cells including CD4^+^ T, CD8^+^ T, and Treg cells, and the PD-L1 expression in circulating immune cells including DC and MDSC cells at different timepoints after radiotherapy.

### Serum Cytokine Assay

The serum was centrifuged at 10,000 rpm for 10 min, and then we diluted the supernatant in 1:2 ratio as sample. Human High Sensitivity Cytokine Premixed Kit A (FCSTM09-08, RandD system, United States) was used to incubate the samples, antibody, and Streptavidin-PE for 3 h, 1 h, and 30 min, respectively. Then serum IL-2, IL-4, IL-5, IL-10, IL-12p, GM-CSF, IFN-γ, and TNF-α were detected using a Luminex 200 system (Luminex Corporation, Austin, TX, United States). What is more, the mixture of standard, blank, and diluted samples was incubated at room temperature for 2 h. Then detection antibody, Streptavidin-HRP, and TMB Subsrate Solution were added and incubated for 1 h, 45 min, and 30 min, respectively. TGF-β1 (#227437–039) and CXCL16 (#309072121) were detected using an ELISA kit (Invitrogen/Thermo Fisher Scientific, United States).

### Statistical Analysis

The experimental data were input and analyzed using SPSS (version 26.0). The survival rate of mice was analyzed by Kaplan–Meier. Continuous variables including tumor growth volumes, counts of immune cells, PD-1/PD-L1 expressions, and cytokine levels were analyzed by one-way ANOVA. All statistical tests were two-sided, and *p* < 0.05 was considered as statistically significant.

## Results

### Tumor Growth and Survival in Lewis Lung Carcinoma Implanted Mouse

**FIGURE 1:**
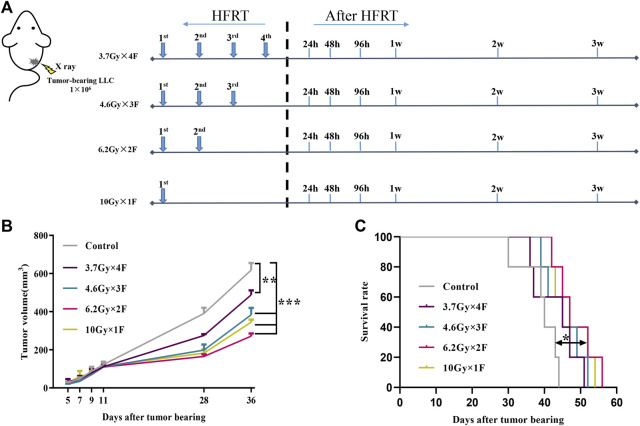
Study design, tumor growth, and survival curves. **(A)** Study design. LLC cells were implanted subcutaneously into the right leg of C57BL/6 mouse on day 0. The mouse received 3.7 Gy × 4 F, 4.6 Gy × 3 F, 6.2 Gy × 2 F, and 10 Gy × 1F radiotherapy. Then collected tail-tip blood samples at different timepoints after radiotherapy. **(B)** Growth curve of tumor in mouse. The tumor volume was significantly reduced in different fractionation radiotherapies. **(C)** Survival curve of mouse. The survival time of 6.2 Gy × 2 F fractionation was significantly prolonged. *p < 0.05, **p < 0.01, ***p < 0.001, compared with the control group.

We observed the tumor volume and survival time of implanted mice after radiotherapy. Tumors occurred at about day 5 after implanting. Mice were irradiated when tumor volume reached about 100 mm^3^. We collected tail-tip blood at the following timepoints: 1 day before radiotherapy and 24 h, 48 h, 96 h, 1 week, 2 weeks, and 3 weeks after radiotherapy (Figure 1A). Tumor growth was significantly delayed in all experimental groups compared with the control group (*p* < 0.01, Figure 1B). Among them, the minimum volume was in the 6.2 Gy × 2 F group (*p* < 0.001, Figure 1B). As for the survival time, there was a significant improvement in the 6.2 Gy × 2 F group compared with the control (*p* < 0.05, Figure 1C).

### Dynamic Changes of Immune Cells in Peripheral Blood in Implanted Mice

**FIGURE 2:**
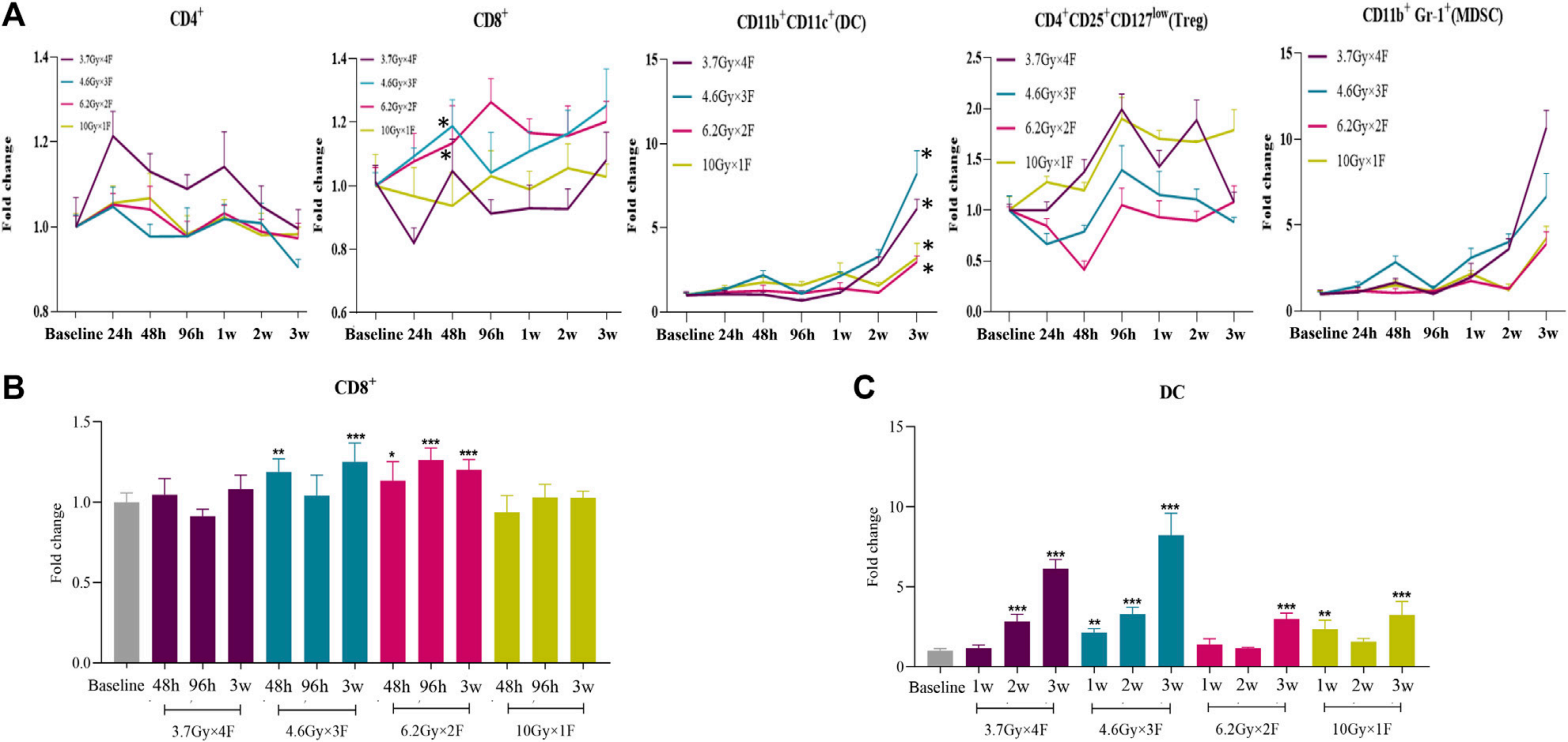
Dynamic changes of immune cell counts in LLC implanted mouse peripheral blood after radiotherapy. **(A)** Trends of CD4^+^ T cells, CD8^+^ T cells, DC, Treg, and MDSC after 4 different fractionation radiotherapies. * The dose in which trend was significant and the time at which difference began. **(B)** Changes of CD8^+^ T cells at 48 h, 96 h, and 3w after radiotherapy, respectively. **(C)** Changes of DC at 1, 2, and 3w after radiotherapy. *p < 0.05,**p < 0.01, ***p < 0.001, compared with baseline.

An increase in the counts of CD4^+^ T cells was identified from 24 h to 1 week after radiotherapy in the 3.7 Gy × 4 F group, but not in other groups (Figure 2A). There was an increase in CD8^+^ T cells from 48 h to 3 weeks after radiotherapy in 4.6 Gy × 3 F and 6.2 Gy × 2 F (*p* < 0.05), but not in 3.7 Gy × 4 F and 10 Gy × 1F (Figures 2A,B). DC counts began to increase from 2 to 3 weeks after radiotherapy in most groups (*p* < 0.01, Figures 2A,C). Treg began to rise at 48 h after radiotherapy in 3.7 Gy × 4 F and returned to baseline at about 3 weeks (Figure 2A). MDSC counts increased from week 2 to week 3 after radiotherapy in all groups (Figure 2A). In the period of 48 h to 2 weeks, the ratio of CD4^+^/Treg and CD8^+^/Treg were 1.41 (0.93–2.51) and 1.62 (1.20–2.73) in 6.2 Gy × 2 F, respectively.

### Expression of PD-1/PD-L1 of Circulating Immune Cells in Implanted Mice

**FIGURE 3:**
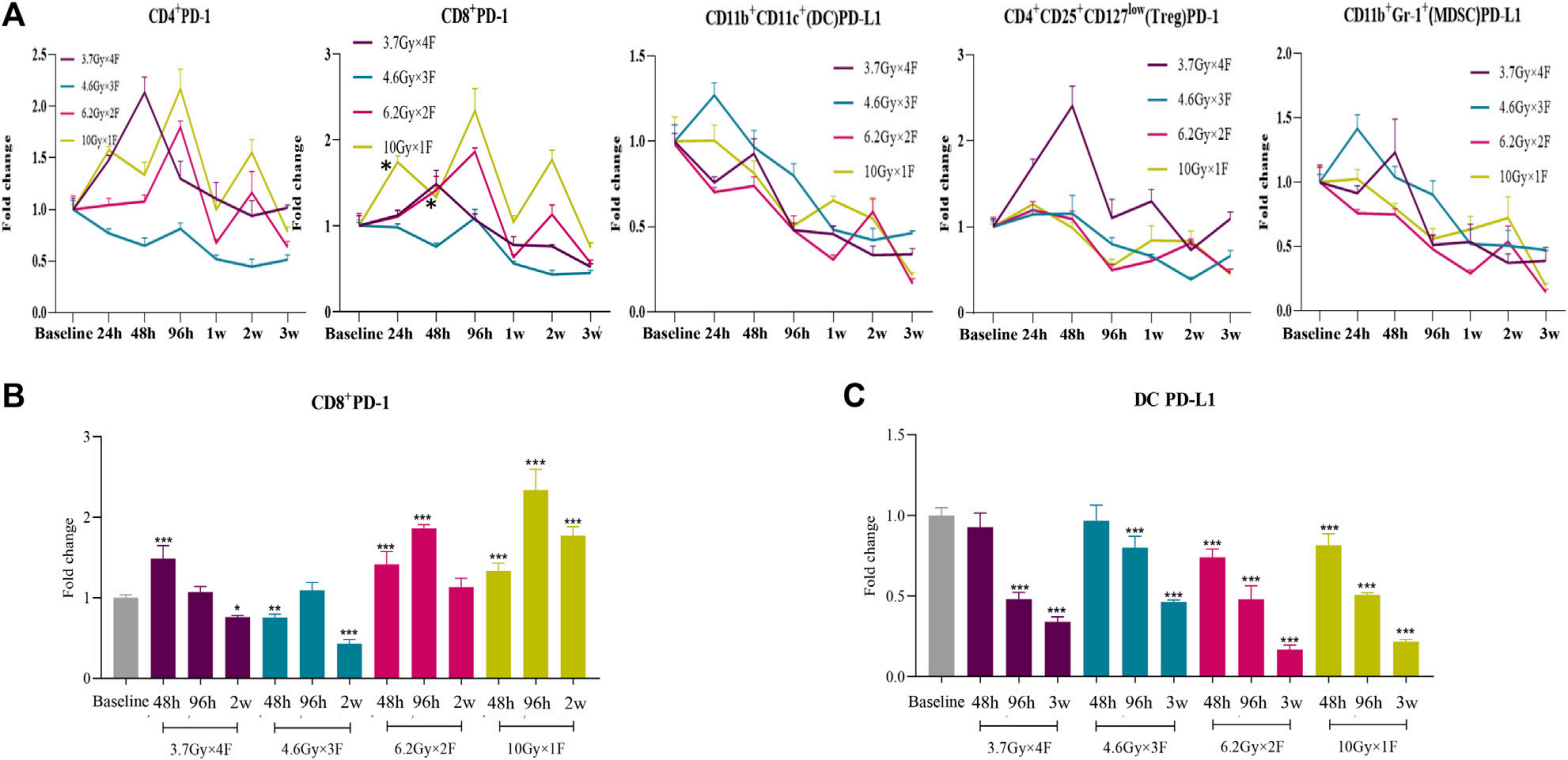
Dynamic trends of PD-1 and PD-L1 expression after radiotherapy in LLC implanted mouse peripheral blood. **(A)** Trends of CD4^+^ PD-1, CD8^+^ PD-1, DC PD-L1, Treg PD-1, and MDSC PD-L1 after 4 different fractionation radiotherapies. * The dose in which trend was significant and the time at which difference began. **(B)** Discrepancy of CD8^+^ PD-1 at 48 h, 96 h, and 2w after radiotherapy, respectively. **(C)** Discrepancy of DC PD-L1 at 48 h, 96 h, and 3w after radiotherapy. *p < 0.05,**p < 0.01, ***p < 0.001, compared with baseline.

CD4^+^ PD-1 and CD8^+^ PD-1 shortly increased from 48 h to 2 weeks after radiotherapy in the 6.2 Gy × 2 F and 10 Gy × 1 F groups (*p* < 0.05, Figures 3A,B). DC PD-L1 gradually decreased from 48 h to 3 weeks after radiotherapy in all experimental groups (*p* < 0.001, Figures 3A,C). Treg PD-1 and MDSC PD-L1 also gradually decreased in most groups (Figure 3A).

### Tumor Immune Microenvironment Changes of Tumor Tissues in Mice

**FIGURE 4:**
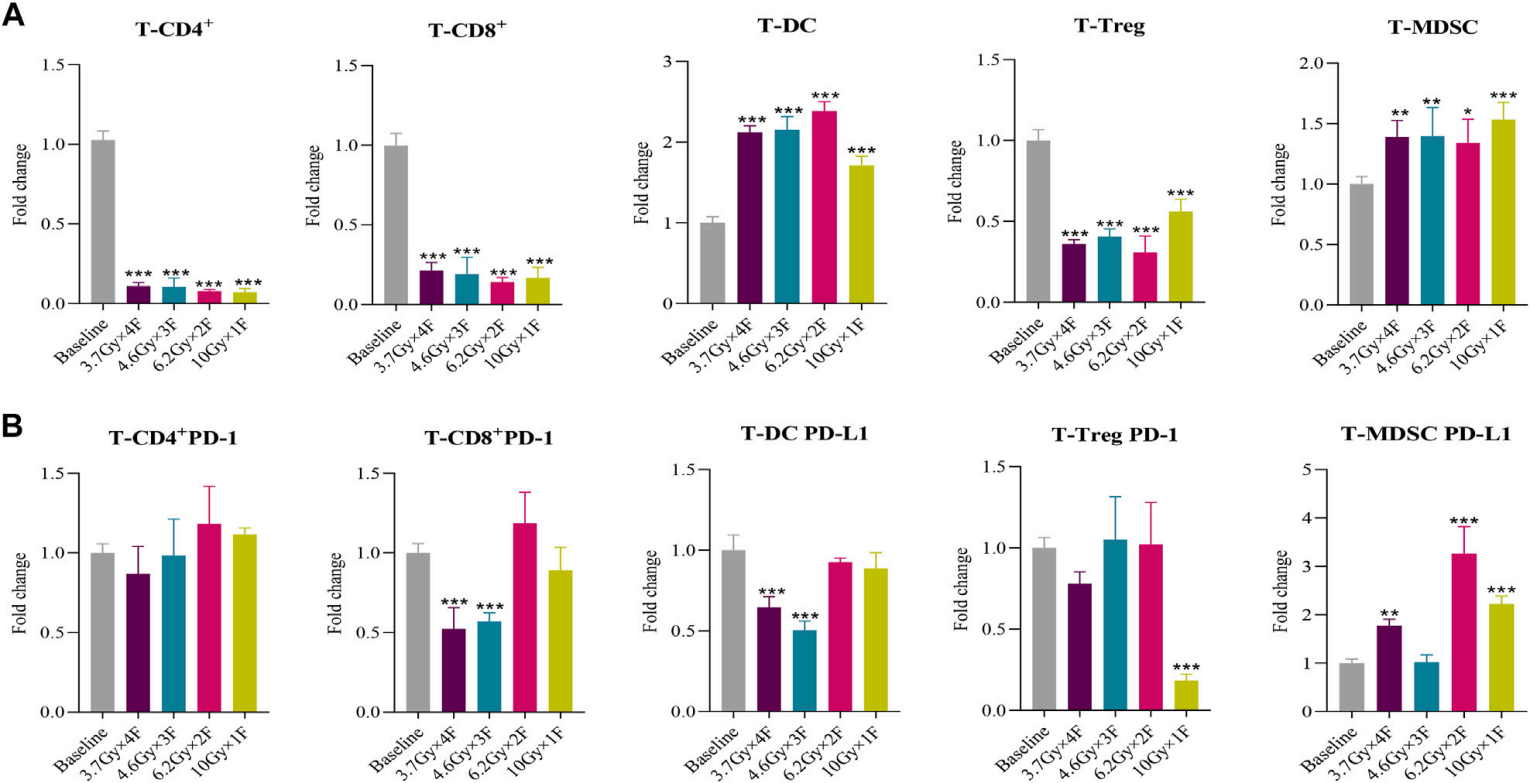
Immune cells and PD-1/PD-L1 changes of tumor tissues in mouse at 48 h after 4 different fractionation radiotherapies. **(A)** Changes of different immune cells at 48 h after radiotherapy in mouse tumor tissues. **(B)** Changes of PD-1 and PD-L1 expression at 48 h after radiotherapy in mouse tumor tissues. *p < 0.05,**p < 0.01, ***p < 0.001, compared with baseline.

The counts of CD4^+^ T cells, CD8^+^ T cells, and Treg decreased at 48 h after radiotherapy in all experimental groups and had an increased proportion of DC and MDSC in tumor tissues (*p* < 0.05, Figure 4A). Both CD8^+^ PD-1 and DC PD-L1 in tumors were downregulated in the 3.7 Gy × 4 F and 4.6 Gy × 3 F groups (*p* < 0.001, Figure 4B). Treg PD-1 decreased at 10 Gy × 1 F, and MDSC PD-L1 was increased except for the 4.6 Gy × 3 F group (*p* < 0.01, Figure 4B). The ratio of CD8^+^ T cells in tumor tissues and peripheral blood was 0.13 (0.10–0.16), and the CD8^+^ PD-1 was 1.03 (0.91–1.17).

### Dynamic Changes of Immune Cells in Human Peripheral Blood

**FIGURE 5:**
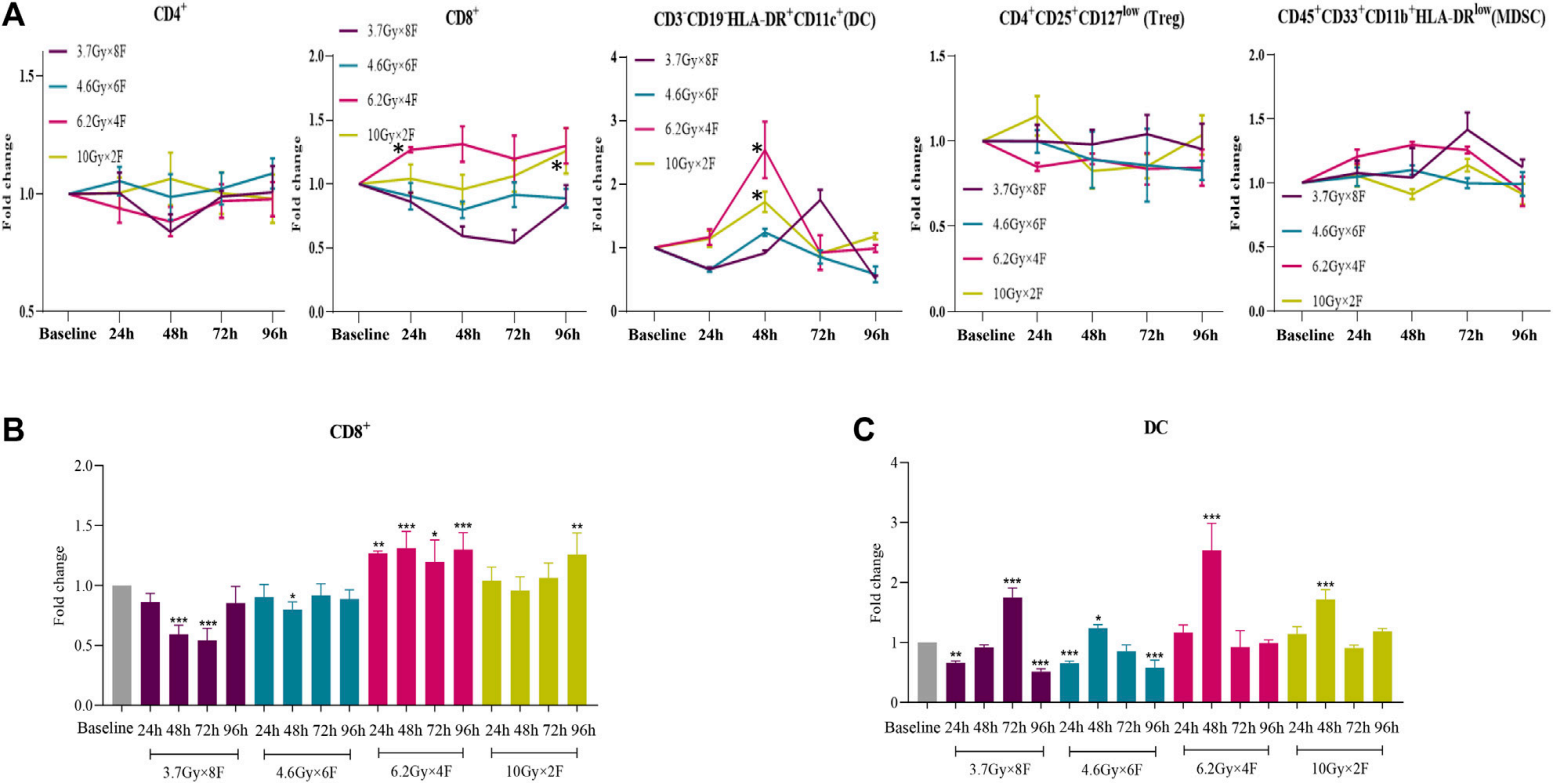
Dynamic changes of different immune cells after radiotherapy in peripheral blood of NSCLC patients. **(A)** Trends of CD4^+^ T cells, CD8^+^ T cells, DC, Treg, and MDSC after 4 different fractionation radiotherapies. * The dose in which trend was significant and the time at which difference began. **(B)** Changes of CD8^+^ T cells at different timepoints after radiotherapy, respectively. **(C)** Changes of DC at different timepoints after radiotherapy.*p < 0.05,**p < 0.01, ***p < 0.001, compared with baseline.

A total of 22 NSCLC patients were recruited from Nov. 1st, 2020 to Aug. 31st, 2021. The clinical characteristics in different groups were collected (Table 1 **)**. The bOR (best overall response) rates of patients with immunotherapy and those without immunotherapy were 53.33 and 42.86%. The mPFS and mOS were 5.59 months (0.92–14.30+) and 6.58 months (1.02–14.30+) in patients with immunotherapy, and mPFS and mOS have not reached (NR) in patients without immunotherapy up to the time of follow-up. CD8^+^ T cells increased from 24 h and maintained a high level to 96 h in 6.2 Gy × 4 F (*p* < 0.05, Figures 5A,B). CD8^+^ T cells also increased in 10 Gy × 2 F at a later timepoint of 96 h (*p* < 0.01, Figures 5A,B). There was an increase in DC cells at 48 h after radiotherapy in 6.2 Gy × 4 F and 10 Gy × 2 F (*p* < 0.001, Figures 5A,C). We did not find dramatic changes in CD4^+^ T cells, Treg, and MDSC in peripheral blood between pre and post-radiotherapy (Figure 5A). From 24 to 96 h, the ratio of CD4^+^/Treg and CD8^+^/Treg were, respectively, 1.09 (0.96–1.17) and 1.43 (1.33–1.50) in 6.2 Gy × 4 F.

**TABLE 1:** Demographics and clinical characteristics in different fractionations of NSCLC patients.

Variables	Total (N = 22)	3.7Gy*8F (n = 5)	4.6Gy*6F (n = 6)	6.2Gy*4F (n = 6)	10Gy*2F (n = 5)
Age	—	—	—	—	—
< 65	12	2	5	3	2
≥65	10	3	1	3	3
Sex	—	—	—	—	—
Male	14	4	2	4	4
Female	8	1	4	2	1
Smoking	—	—	—	—	—
Yes	14	4	3	4	3
No	8	1	3	2	2
Pathology	—	—	—	—	—
ADC	14	2	4	5	3
SCC	8	3	2	1	2
T stage	—	—	—	—	—
T1	1	0	0	1	0
T2	4	1	0	1	2
T3	4	2	2	0	0
T4	13	2	4	4	3
N stage	—	—	—	—	—
N0	1	0	0	0	1
N1	1	0	1	0	0
N2	6	1	0	4	1
N3	14	4	5	2	3
M stage	—	—	—	—	—
M0	0	0	0	0	0
M1	22	5	6	6	5
Concurrent chemotherapy	—	—	—	—	—
Yes	6	2	1	2	1
No	16	3	5	4	4
Concurrent immunotherapy	—	—	—	—	—
Yes	15	3	4	4	4
Pembrolizumab	3	2	0	0	1
Nivolumab	1	1	0	0	0
Atezolizumab	1	0	1	0	0
Tislelizumab	2	0	1	1	0
Toripalimab	4	0	1	1	2
Camrelizumab	3	0	0	2	1
Sintilimab	1	0	1	0	0
No	7	2	2	2	1
Target gene mutation	—	—	—	—	—
Yes	7	0	2	3	2
EGFR	4	0	1	1	2
ALK	1	0	1	0	0
KRAS	1	0	0	1	0
BRAF	1	0	0	1	0
No	15	5	4	3	3

ADC, adenocarcinoma; SCC, squamous cell carcinoma.

### Expression of PD-1/PD-L1 of Circulating Immune Cells in Patients

**FIGURE 6:**
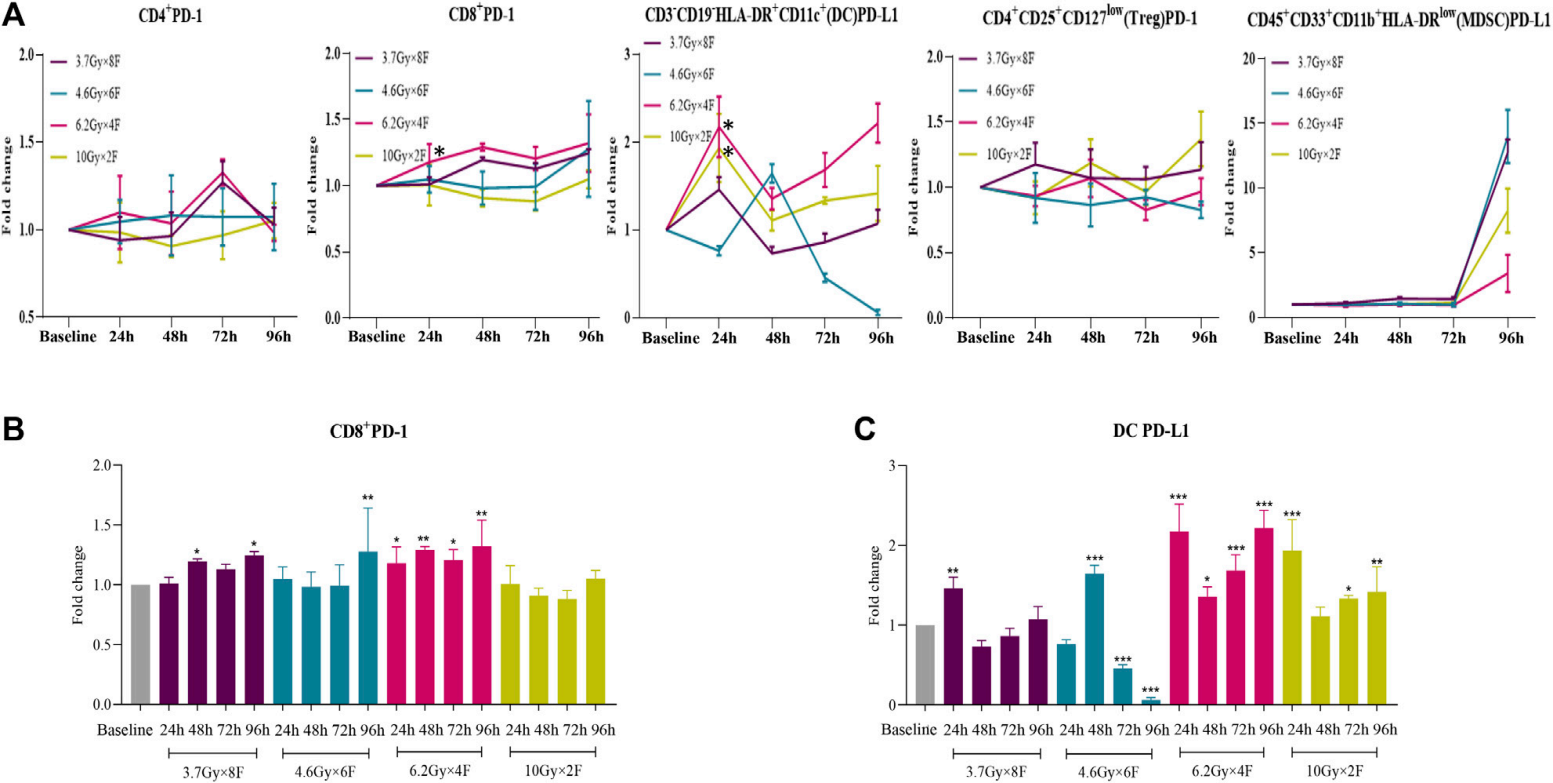
Dynamic changes of PD-1 and PD-L1 expression after radiotherapy in peripheral blood of NSCLC patients. **(A)** Trends of CD4^+^ PD-1, CD8^+^ PD-1, DC PD-L1, Treg PD-1, and MDSC PD-L1 after 4 different fractionation radiotherapies. * The dose in which trend was significant and the time at which difference began. **(B)** Discrepancy of CD8^+^ PD-1 at different timepoints after radiotherapy, respectively. **(C)** Discrepancy of DC PD-L1 at different timepoints after radiotherapy.*p < 0.05,**p < 0.01, ***p < 0.001, compared with baseline.

There were significant increases in CD8^+^ PD-1 from 24 to 96 h after radiotherapy in 6.2 Gy × 4 F, from 48 h in 3.7 Gy × 8 F, and at 96 h in 4.6 Gy × 6 F, respectively (*p* < 0.05, Figures 6A,B). DC PD-L1 significantly increased from 24 to 96 h in 6.2 Gy × 4 F and 10 Gy × 2 F except for the timepoint of 48 h (*p* < 0.05, Figures 6A,C). There were no obvious changes in CD4^+^ PD-1 and Treg PD-1 between pre and post-radiotherapy at most timepoints of the experimental groups. MDSC PD-L1 increased at 96 h in all experimental groups (Figure 6A).

### Detection of Cytokines in Serum of Patients

**FIGURE 7:**
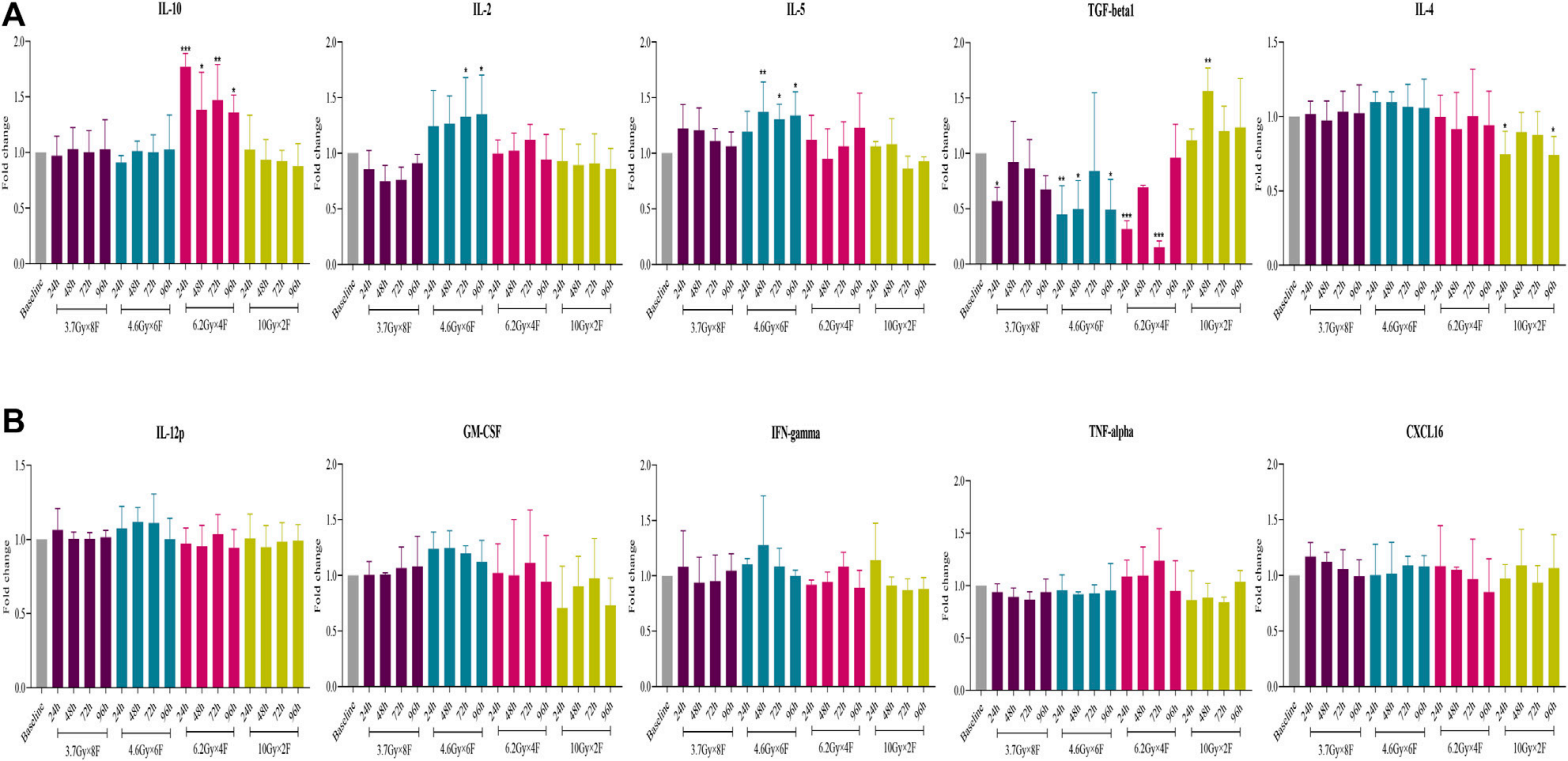
Changes of cytokine expression after radiotherapy in peripheral blood of NSCLC patients. **(A)** Expression of IL-10, IL-2, IL-5, TGF-β1, and IL-4 had different changes after 4 fractionation radiotherapies. **(B)** No significant change on IL-12p, GM-CSF, IFN-γ, TNF-α, and CXCL16 between pre and post-radiotherapy. *p < 0.05,**p < 0.01, ***p < 0.001, compared with baseline.

There was a significant increase in IL-10 from 24 to 96 h after radiotherapy in 6.2 Gy × 4 F (*p* < 0.05) and an increase in IL-2 and IL-5 in 4.6 Gy × 6 F (*p* < 0.05, Figure 7A). On the contrary, there was a significant decrease in TGF-β1 at different timepoints in 3.7 Gy × 8 F, 4.6 Gy × 6 F, and 6.2 Gy × 4 F (*p* < 0.05) and a decrease in IL-4 at 24 and 96 h in 10 Gy × 2 F (*p* < 0.05, Figure 7A). There were no obvious changes in IL-12p, GM-CSF, IFN-γ, TNF-α, and CXCL16 between pre and post-radiotherapy in all the groups (Figure 7B).

## Discussion

Radiotherapy can achieve a synergistic effect with immunotherapy by recruiting T cells to the irradiated tumor area and increasing the vulnerability of tumor cells to T cells (Galluzzi et al., 2017). Nevertheless, how to determine the optimal combination strategy of radiotherapy and immunotherapy remains an unsolved problem in clinical practice.

As regarded, timing between radiotherapy and immunotherapy as well as the fractionations are important considerations. Studies have shown that the timing of radiotherapy combined with ICIs depends on different types of tumors and ICIs (Young et al., 2016; Lesueur et al., 2018). Therefore, there is controversy about the optimal timepoints of ICIs and radiotherapy. Most studies have revealed that the comprehensive immune effect is positive and CD8^+^ T cells play a vital role in HFRT and SBRT (Reits et al., 2006; Lee et al., 2009). CD8^+^ T cells residing in tumors are mainly a group of high proliferation capability and exhausted function, which cannot effectively kill tumor cells (Li et al., 2019; Sanmamed et al., 2021). After HFRT, CD8^+^ T cells in peripheral blood migrate to tumors, kill tumor cells, and further activate DCs, thereby turning “cold” tumors into “hot” tumors. The effect of PD-1 inhibitors depends on the intratumoral infiltration of CD8^+^ T cells derived from peripheral blood (Huang et al., 2017; Yost et al., 2019). In this study, we found that immune cells, especially CD8^+^ T cells, in mice tumor tissues and peripheral blood showed a time-dependent dynamic change after radiotherapy. CD8^+^ T counts increased from 48 h to 3 weeks in 4.6 Gy × 3 F and 6.2 Gy × 2 F, and CD8^+^ PD-1 shortly increased from 48 h to 2 weeks in 6.2 Gy × 2 F and 10 Gy × 1 F. These results indicate that 48 h after HFRT may be a critical timepoint for immune response. Then we selected the timepoint of 48 h to verify in mouse tumor tissue and found that CD8^+^ T cells were not increased yet, indicating that the immune system has been activated in blood within 48 h after radiotherapy, earlier than in tumor tissue, which was consistent with the research by Matsumura (Matsumura et al., 2008). Besides, in peripheral blood of NSCLC patients, CD8^+^ T cells and CD8^+^ PD-1 were increased significantly from 24 to 96 h after radiotherapy. Furthermore, DC cells began to be significantly increased and continued to rise until 3 weeks in mice, and there was a similar trend in NSCLC patients before 96 h. DC PD-L1 showed a high level from 24 to 96 h in patients. On the other hand, MDSCs, which were regarded as suppressors of the immune cell response, increased from 2 weeks and continued to rise until 3 weeks in mouse peripheral blood. Besides, we also compared the ratio of CD4^+^/Treg and CD8^+^/Treg, respectively. All the ratios were consistent with the cell counts.

Collectively, the rule of changes in our study suggests that intratumoral infiltrating T cells being induced after local radiotherapy may be derived from the mobilization and chemotaxis of the systemic immune system. We speculate that CD8^+^ T cells are mobilized fully in the peripheral blood from 48 h to 2 weeks after radiotherapy, preferentially recruited and activated into tumor tissue; in that period, the efficacy of radiotherapy will be enhanced when combined with ICIs. This result is consistent with the PACIFIC study in which the timing of intervention ICIs is within 14 days. Most studies have shown that early interventional immunotherapy after radiotherapy has better efficacy (Shaverdian et al., 2017; Aggarwal et al., 2019; Theelen et al., 2019), and only a retrospective study reported at the 2019 ASCO meeting showed that immunotherapy given at 3 weeks after SBRT has better OS (Wegner et al., 2019). Using evidence-based medicine, the subgroup analysis of randomized controlled studies is more reliable than that of retrospective analysis. Therefore, it is possible that a shorter interval of immunotherapy after radiotherapy leads to better effect. However, due to the high single dose of SBRT or HFRT, synchronization immunotherapy or premature use of ICIs after radiotherapy may cause an increase in side effects. Our results are generally consistent with previous research, but differ slightly in the specific timepoints. We speculate that the difference may be due to race, number of cases, type of ICIs, and fractionation dose. In future, the optimal timepoints for combining radiotherapy with immunotherapy need further study.

When radiotherapy is combined with ICIs, the fractionation dose is another key factor for optimal outcome. Dewan et al. (Dewan et al., 2009) used the TSA breast cancer cell model and the MCA38 colorectal cancer model and found that when combined with CLTA-4 inhibitors, 8 Gy × 3F was significantly better than 6 Gy × 5 F or 12 Gy × 1 F regardless of local tumor control or abscopal immune response, suggesting that different fractionations also have different effects on the cancer therapy when combined with ICIs. In this study, the tumor volume was significantly reduced after radiotherapy with 4 different fractionations of the same BED. Moreover, the longer survival time appeared after radiotherapy of 6.2 Gy × 2 F. A clinical trial in the United Kingdom demonstrated the similar results that different fractionation doses with similar BED could cause different efficacy and prognosis of tumors (The et al., 2008). On the other hand, we found that CD8^+^ T cells were significantly increased in 6.2 Gy × 2 F, along with CD8^+^ PD-1 being increased in 6.2 Gy × 2 F fractionation in the peripheral blood of mice. In peripheral blood of NSCLC patients, CD8^+^ T cells and CD8^+^ PD-1 maintained a high level in 6.2 Gy × 4 F, and CD8^+^ T cells also increased in 10 Gy × 2 F. Besides, DC cells began to be significantly increased after radiotherapy in all fractionations in mice. In peripheral blood of NSCLC patients, we discovered that DC cells significantly increased and hit a small peak in 6.2 Gy × 4 F and 10 Gy × 2 F and then gradually returned to the baseline level, being accompanied by a high expression of DC PD-L1 in 6.2 Gy × 4 F and 10 Gy × 2 F.

Chen et al. (Welsh et al., 2020a) conducted a phase I/II randomized clinical trial comparing the efficacy of paprizumab alone versus combination with conventional fractionated radiotherapy (45Gy/15F) or SBRT (50Gy/4F) in the treatment of advanced NSCLC. The mPFS of the paprizumab + SBRT group was significantly better than that of the paprizumab + conventionally fractionated radiotherapy group (9.1 months vs*.* 5.1 months). In addition, Baas et al. (Welsh et al., 2020b) analyzed pembro-RT and MDACC studies and found that the combination of pabulizumab with ablative radiotherapy (24Gy/3F and 50Gy/4F) had a better ORR than non-ablative radiotherapy (45Gy/15F) and the pabulizumab alone group. Therefore, in terms of fractionation dose, studies tend to support SBRT or HFRT combined with immunotherapy, which can enhance the antitumor effect more than conventional fractionated radiotherapy. Here, we demonstrate that 6.2 and 10 Gy would be better doses of HFRT, which will produce an optimal immunoactivated status from 48 h after radiotherapy and the immune synergistic effect may be maximized when combined with ICIs. At these doses, both numbers and PD-1 expression of positive immune cells increase in TIME, which will be beneficial for ICIs in NSCLC. There is a reminder that the best timing of immune intervention could be 96 h later under 10 Gy fractionation, especially in two but not single doses.

Due to the limit of tail vein blood in each mouse, we detected the immune cells in tumor tissues instead of cytokines. Likewise, we detected cytokines in NSCLC patients instead of immune cells in tumor tissues. Interleukin-10 (IL-10) was discovered as an anti-inflammatory factor. However, increasing evidence revealed IL-10 can induce antitumor effects in an immune-dependent manner, which indicates that it also plays a bidirectional role in immune regulation of tumors (Sato et al., 2011). Studies have demonstrated that effector T cells were the main source of IL-10, and IL-10 can promote CD8^+^ T cell responses by binding to IL-10 receptor (Fujii et al., 2001; Wilke et al., 2011). Besides, Qiao (Qiao et al., 2019) generated a Cetuximab-based IL-10 fusion protein (CmAb-(IL10)2) and found that it could prevent CD8^+^ tumor-infiltrating lymphocyte apoptosis, further revealing that IL-10 could potentiate CD8^+^ T cell-mediated antitumor immunity. In this study, IL-10 was increased significantly from 24 to 96 h, when the number of CD8^+^ T cells and CD8^+^ PD-1 expression were also increased after 6.2G×4F radiotherapy in human peripheral blood. The result revealed that HFRT can induce CD8^+^ T cells to produce IL-10, which enhances the proliferation, differentiation, activity, and function of CD8^+^ T cells, especially in 6.2 Gy. In the TGF family, TGF-β1 is the most widely distributed in the immune system, and with the development of tumors, it can continuously promote the invasion, metastasis, and deterioration. Marie confirmed that TGF-β1 mainly plays a role in Tregs induction by regulating Foxp3 positive expression in Tregs (Marie et al., 2005). Furthermore, convincing evidence has verified that IL-4 directly acts on CD8^+^ T cells, reduces or even eliminates its cytotoxicity, and promotes the infiltration of Treg into tumors, thus establishing the immunosuppressive state and promoting tumor growth by avoiding the recognition of the immune system (Wynn, 2003). Here, we found that TGF-β1 decreased significantly in 3.7 Gy × 8 F, 4.6 Gy × 6 F, and 6.2 Gy × 4 F and that IL-4 decreased in 10 Gy × 2 F after radiotherapy, revealing that HFRT also achieves antitumor immunity by reducing TGF-β1 and IL-4, thus contributing to regulating Tregs and CD8^+^ T cell function in TIME. Besides, IL-2 and IL-5, which are regarded as positive immune regulators, are also found to increase significantly in 4.6 Gy × 6 F. Therefore, we find different cytokines change in different fractionations and speculate that the immune effect of different fractionation radiotherapies may be related to different target cytokines and induce an immune activation state after HFRT.

In the part of clinical data, we provided the bOR, mPFS, and mOS. However, there are many impact factors in this clinical practice, such as the baseline of patients, the number of treatment lines, and the different duration of immunotherapy. We do not think the efficacy and survival for the patients with or without immunotherapy are important results. Moreover, most of the clinical cases who received immunotherapy were not in the time window of 48 h to 2 weeks because we did not have any conclusion before this work.

To the best of our knowledge, this study is the first to prove the positive comprehensive immune effect on the timing and fractionation dose of HFRT. CD8^+^ T cells are the most important indicator of TIME, so we took CD8^+^ T cells as the main effector cells to judge the immune response. CD8^+^ T increased after HFRT both in peripheral blood of NSCLC patients and mouse models, revealing that HFRT can induce a positive immune response which may be beneficial for ICIs. Comprehensively considering the results of CD8^+^ T cells, DC cells, and cytokines, 3.7 and 4.6Gy are not recommended as the preferred fractionations compared with 6.2 and 10Gy. However, this is just our preliminary result. We are designing another animal experiment and prospective study based on our current findings and carry out clinical trials with larger samples for verification in the future.

In spite of the major strength of validating the changes of TIME after HFRT in both animal and human experiments, this study still has several limitations. First, taking into account clinical treatment efficacy, we adopted different fractionation doses between mouse and human. Second, we only choose LLC cells to establish the implanted mice model because it is not suitable to use xenografts derived from human cancer cells in immunodeficient mice to explore the immune microenvironment. Third, there were some other combination treatments during radiotherapy in different groups in patients. Finally, we did not perform cell isolation to detect cytokine secretion in tumor tissue and cell killing experiments *in vitro*.

## Conclusion

In summary, HFRT induces the increase in CD8^+^ T cells and positive immune cytokine response in specific periods and fractionation doses. It was the optimal time window from 48 h to 2 weeks for immune response, especially in 6.2 Gy fractionation. The best immune response was 96 h later in 10 Gy fractionation, delivered twice instead of a single dose. During this time window, the intervention of immunotherapy may achieve a better effect. Future work should include exploration of the relationship between radiotherapy and TIME deeply.

## Data Availability

The original contributions presented in the study are included in the article/Supplementary Material; further inquiries can be directed to the corresponding author.
